# Supplementary material for: Complexity of the COVID-19 pandemic in Maringá
Source: Sci Rep. 2023 Aug 4;13:12695. doi: 10.1038/s41598-023-39815-x (PMC10403588; doi:10.1038/s41598-023-39815-x)
Supplement: Supplementary file 1 — Supplementary Information. [file 41598_2023_39815_MOESM1_ESM.pdf]

Complexity of the COVID-19 pandemic in Maringá

Andre S. Sunahara[1], Arthur A. B. Pessa[1], Matjaž Perc[2,3,4,5,6,*], and Haroldo V. Ribeiro[1,†]

[1]Departamento de Física, Universidade Estadual de Maringá - Maringá, PR 87020-900, Brazil
[2]Faculty of Natural Sciences and Mathematics, University of Maribor, Koroška cesta 160, 2000 Maribor, Slovenia
[3]Department of Medical Research, China Medical University Hospital, China Medical University, Taichung, Taiwan
[4]Alma Mater Europaea, Slovenska ulica 17, 2000 Maribor, Slovenia
[5]Department of Physics, Kyung Hee University, 26 Kyungheedae-ro, Dongdaemun-gu, Seoul, Republic of Korea
[6]Complexity Science Hub Vienna, Josefstädterstraße 39, 1080 Vienna, Austria
[*]email: matjaz.perc@gmail.com
[†]email: hvr@dfi.uem.br

Supplemental Materials

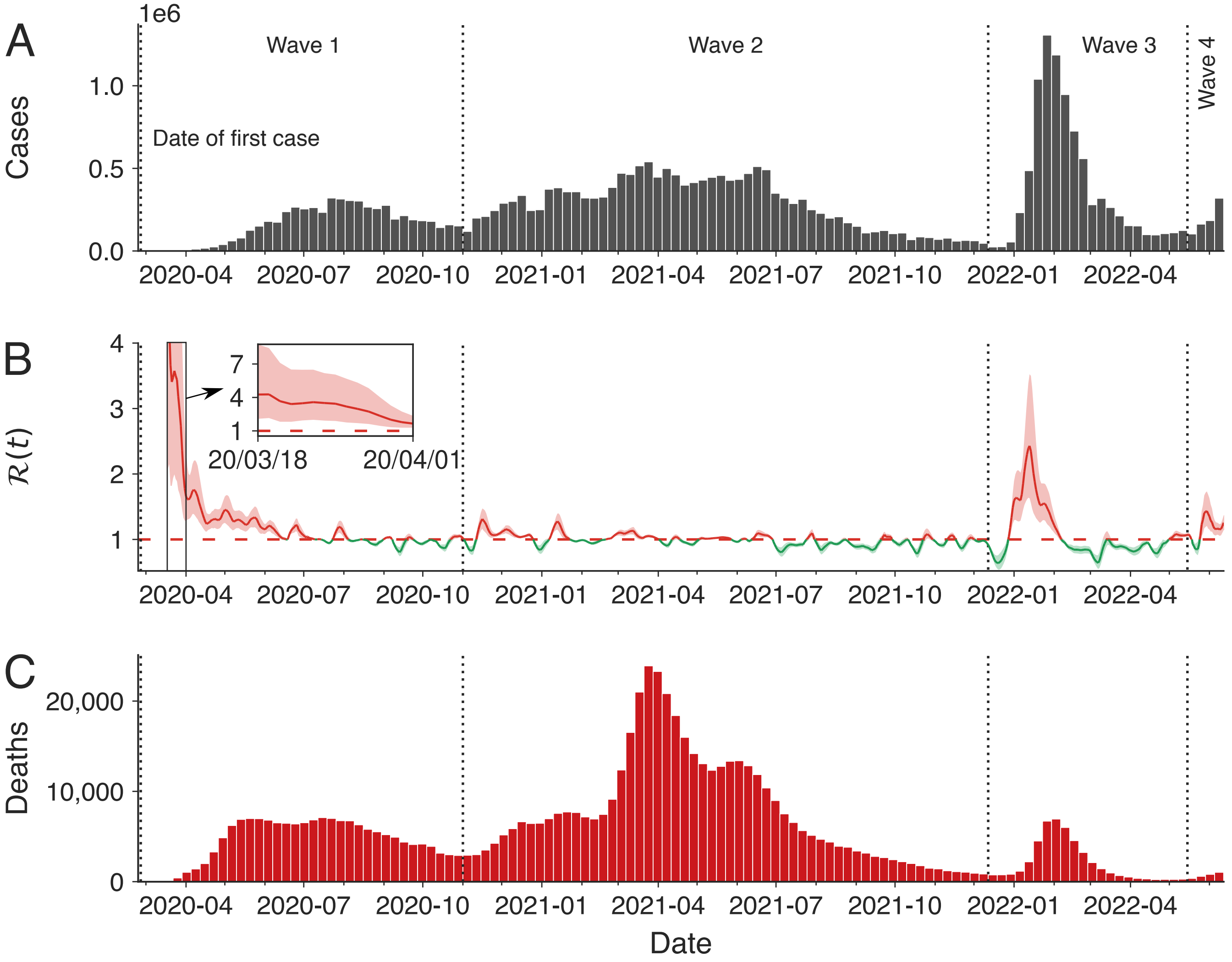

Figure S1. Indicators of the COVID-19 pandemic in Brazil. (A) Weekly numbers of confirmed cases of COVID-19 between 25 February 2020 and 12 June 2022. (B) Instantaneous reproduction number $\mathscr{R}(t)$ from 18 March 2020 to 12 June 2022. Shaded regions represent the 95% confidence intervals, and the dashed horizontal line indicates the epidemic threshold $\mathscr{R}(t) = 1$. (C) Weekly COVID-19 death toll between 25 February 2020 and 12 June 2022. In all panels, vertical dashed lines delineate the four identified waves of COVID-19 cases.

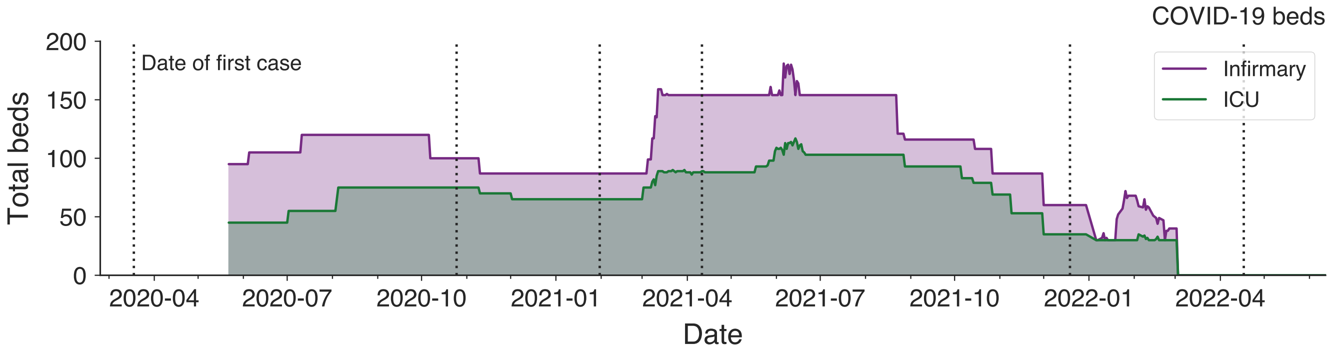

Figure S2. Evolution of the total number of hospital beds exclusively dedicated to COVID. Daily number of the total infirmary (purple curve) and intensive care (green curve) beds exclusively available for COVID-19 patients in Maringá from 22 May 2020 to 12 June 2022. Vertical dashed lines delineate the six identified waves of COVID-19 cases.

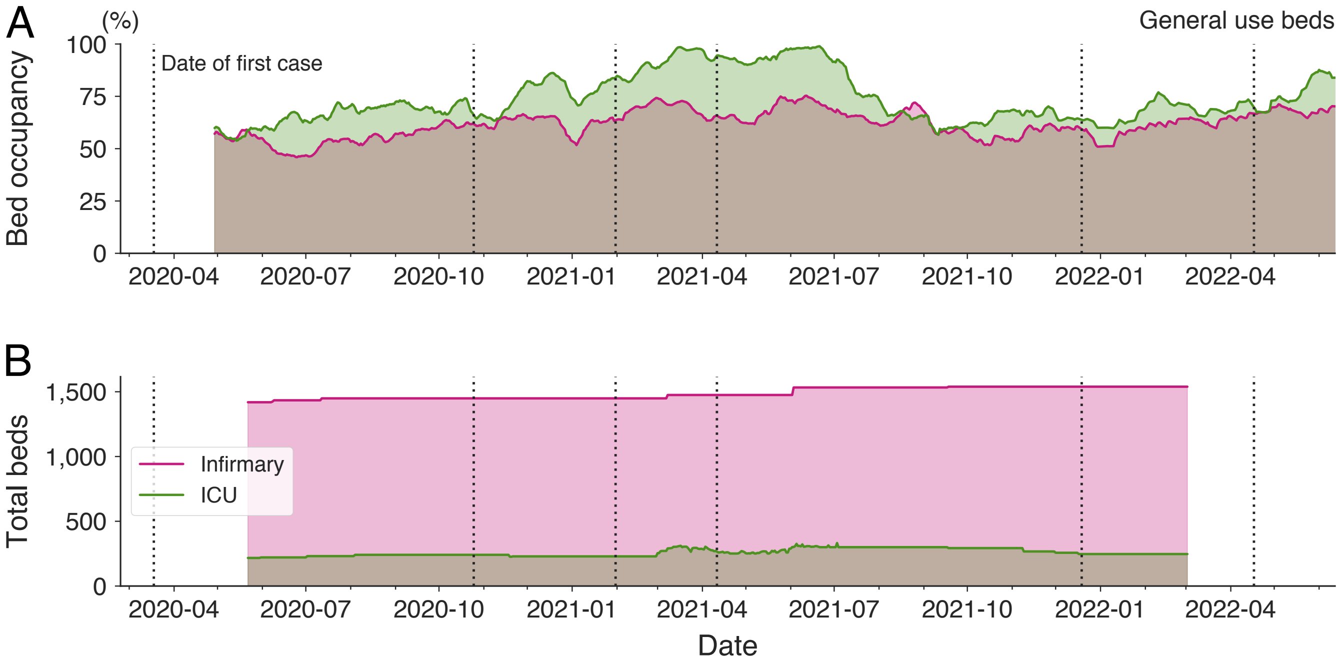

Figure S3. Occupancy infirmary and intensive care beds for general use. (A) Daily percentage occupancy of infirmary (purple curve) and intensive care (green curve) beds available for general use in Maringá from 29 April 2020 to 12 June 2022. The curves correspond to 7-day moving averages. (B) Daily number of the total infirmary (purple curve) and intensive care (green curve) beds for general use in Maringá from 22 May 2020 to 2 March 2022. In both panels, vertical dashed lines delineate the six identified waves of COVID-19 cases.

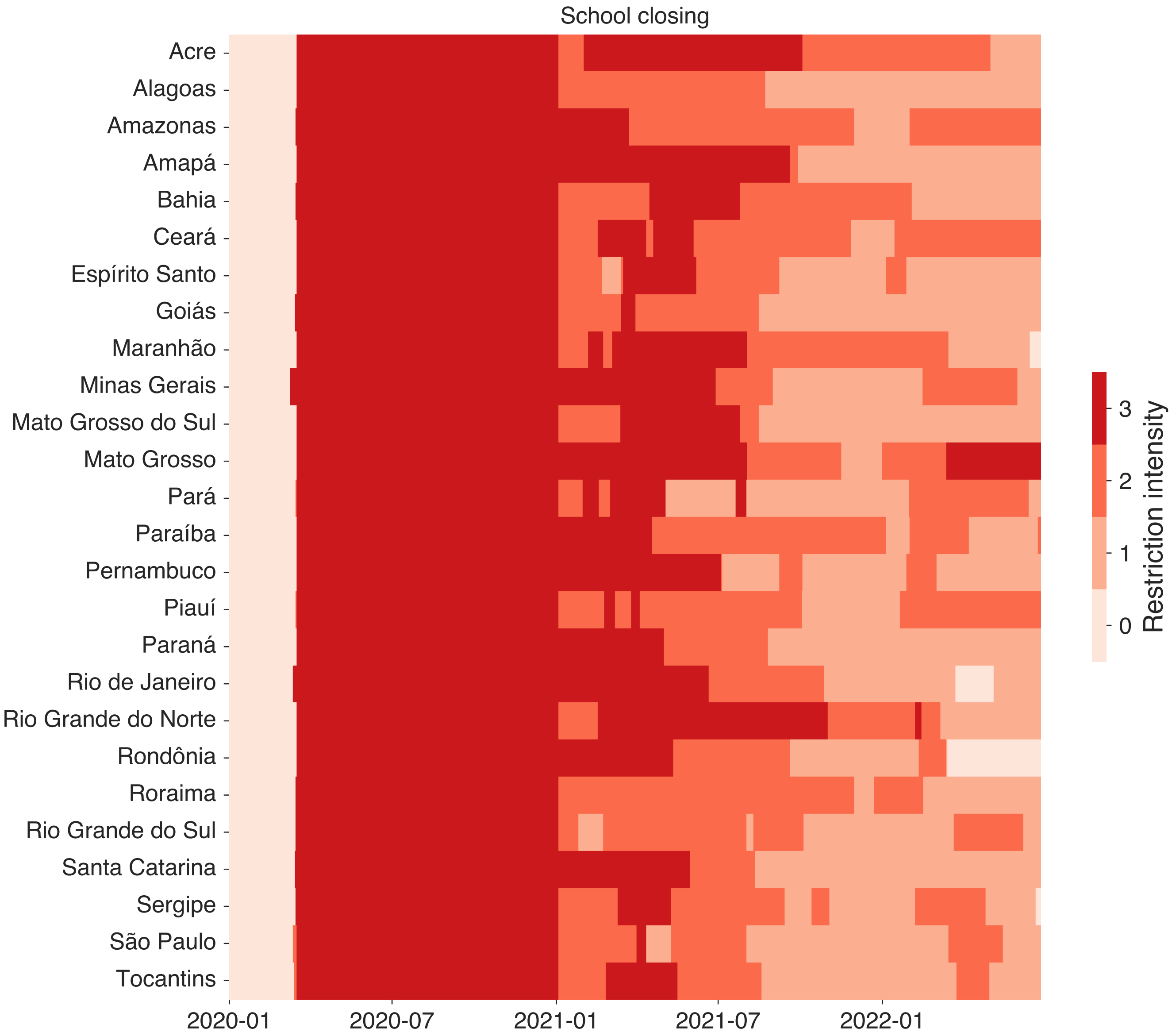

Figure S4. School closing non-pharmaceutical interventions among the Brazilian states. The heatmap displays the temporal evolution of the implementation of school closing interventions for all Brazilian states from January 2020 to June 2022. The color code stands for the degree of restriction: "no measures" (0), "recommend closing or all schools open with alterations resulting in significant differences compared to non-COVID-19 operations" (1), "require closing (only some levels or categories, such as just high school or just public schools)" (2), and "require closing all levels" (3).

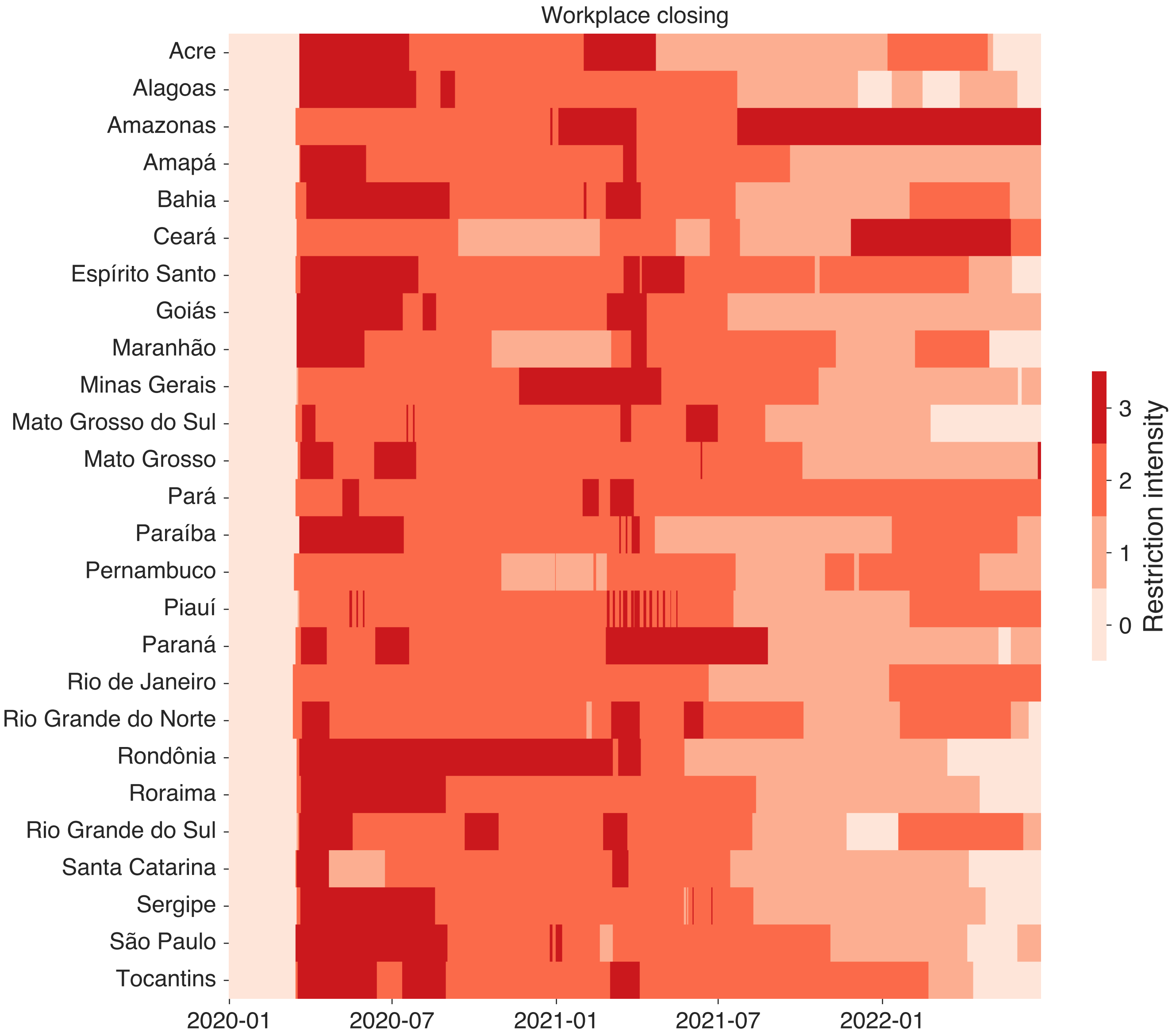

Figure S5. Workplace closing non-pharmaceutical interventions among the Brazilian states. The heatmap displays the temporal evolution of the implementation of workplace closing interventions for all Brazilian states from January 2020 to June 2022. The color code stands for the degree of restriction: “no measures” (0), “recommend closing (or recommend work from home) or all businesses open with alterations resulting in significant differences compared to non-COVID-19 operation” (1), “require closing (or work from home) for some sectors or categories of workers” (2), and “require closing (or work from home) for all-but-essential workplaces (e.g., grocery stores and doctors)” (3).

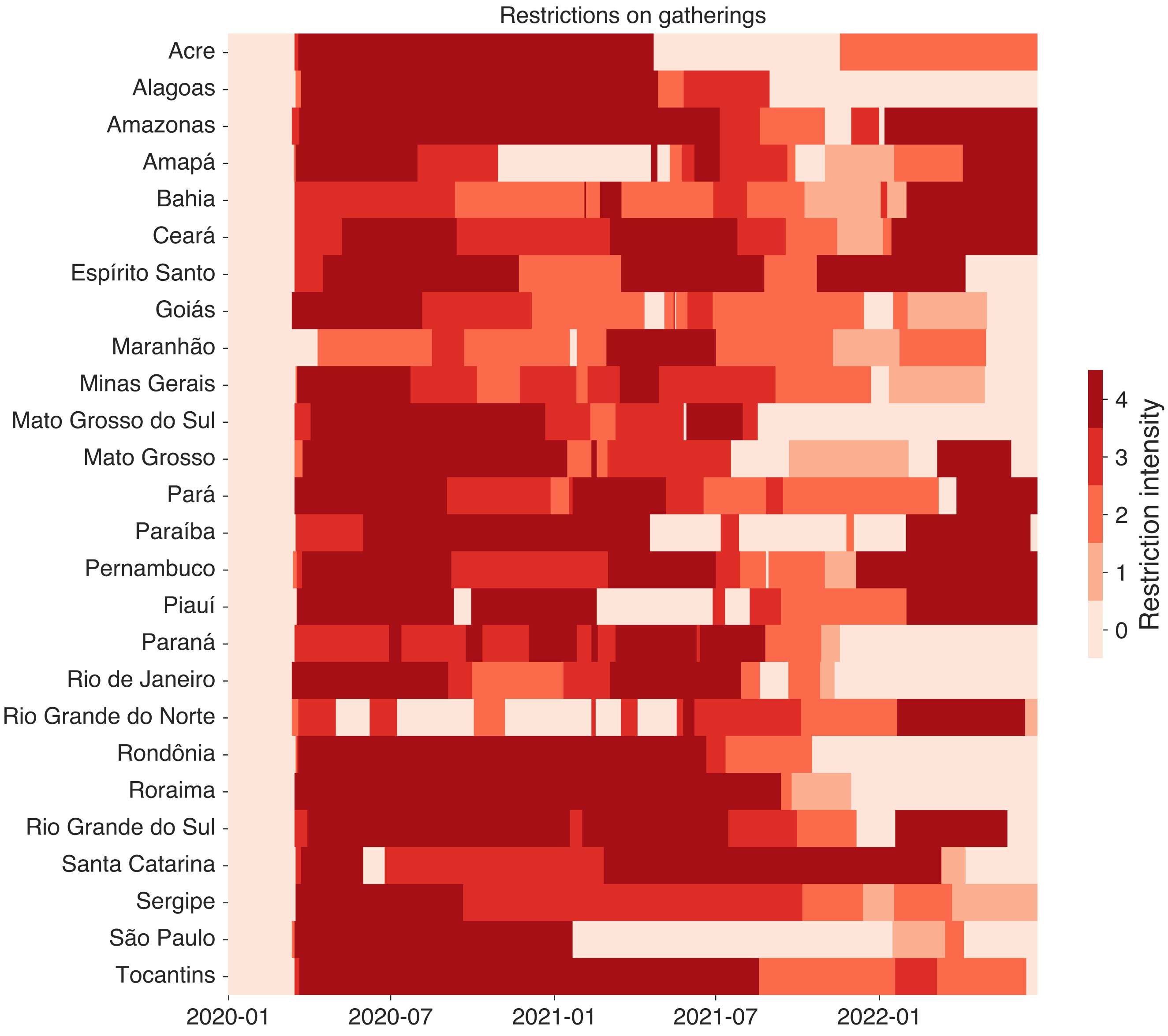

Figure S6. Restrictions on gatherings among the Brazilian states. The heatmap displays the temporal evolution of the implementation of restriction on gatherings interventions for all Brazilian states from January 2020 to June 2022. The color code stands for the degree of restriction: “no restrictions” (0), “restrictions on very large gatherings (the limit is above 1000 people)” (1), “restrictions on gatherings between 101-1000 people” (2), “restrictions on gatherings between 11-100 people” (3), and “restrictions on gatherings of 10 people or less” (4).

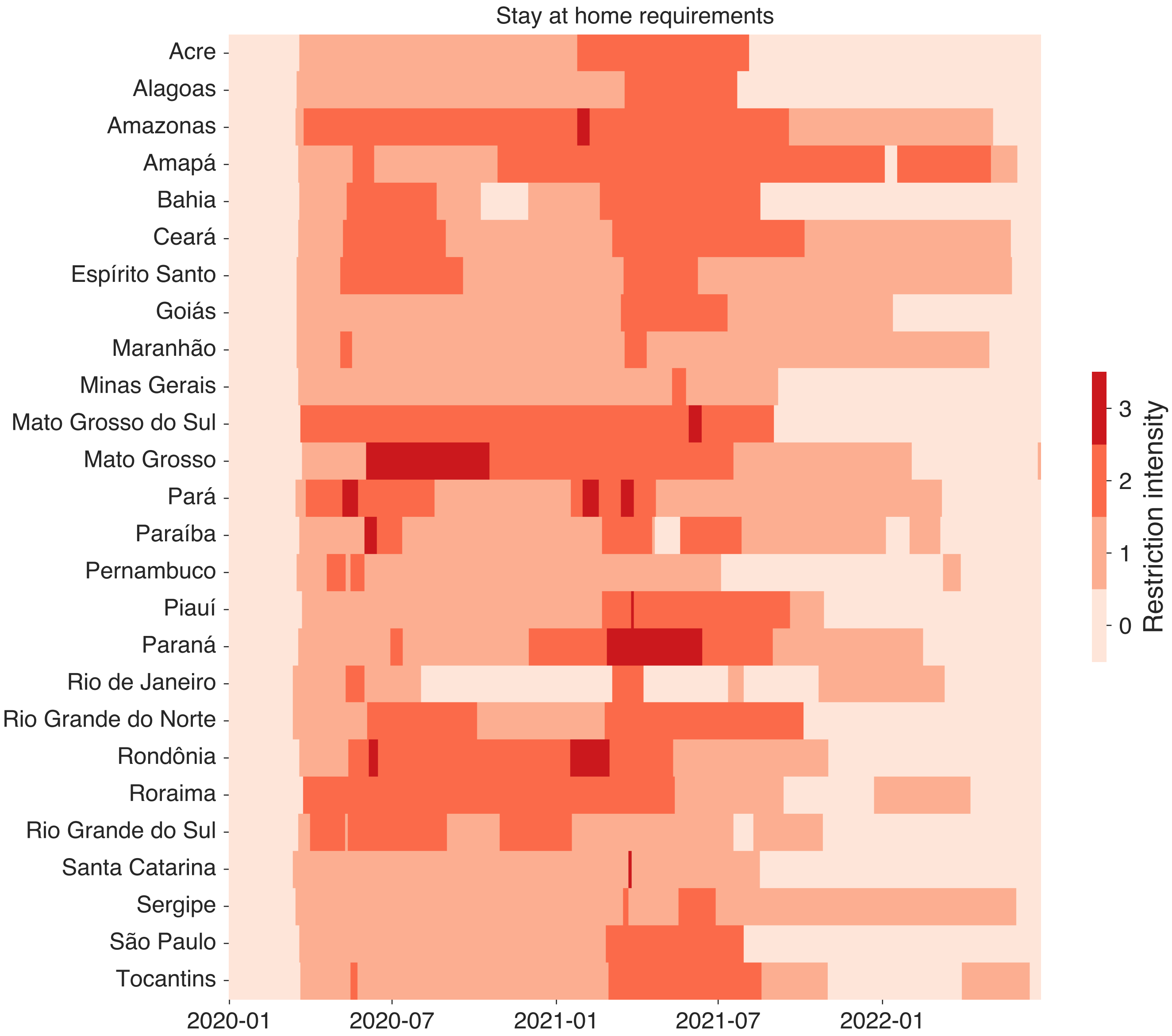

Figure S7. Stay at home requirements among the Brazilian states. The heatmap displays the temporal evolution of the implementation of stay at home interventions for all Brazilian states from January 2020 to June 2022. The color code stands for the degree of restriction: "no measures" (0), "recommend not leaving house" (1), "require not leaving house with exceptions for daily exercise, grocery shopping, and 'essential' trips" (2), and "require not leaving house with minimal exceptions (e.g., allowed to leave once a week or only one person can leave at a time)" (3).

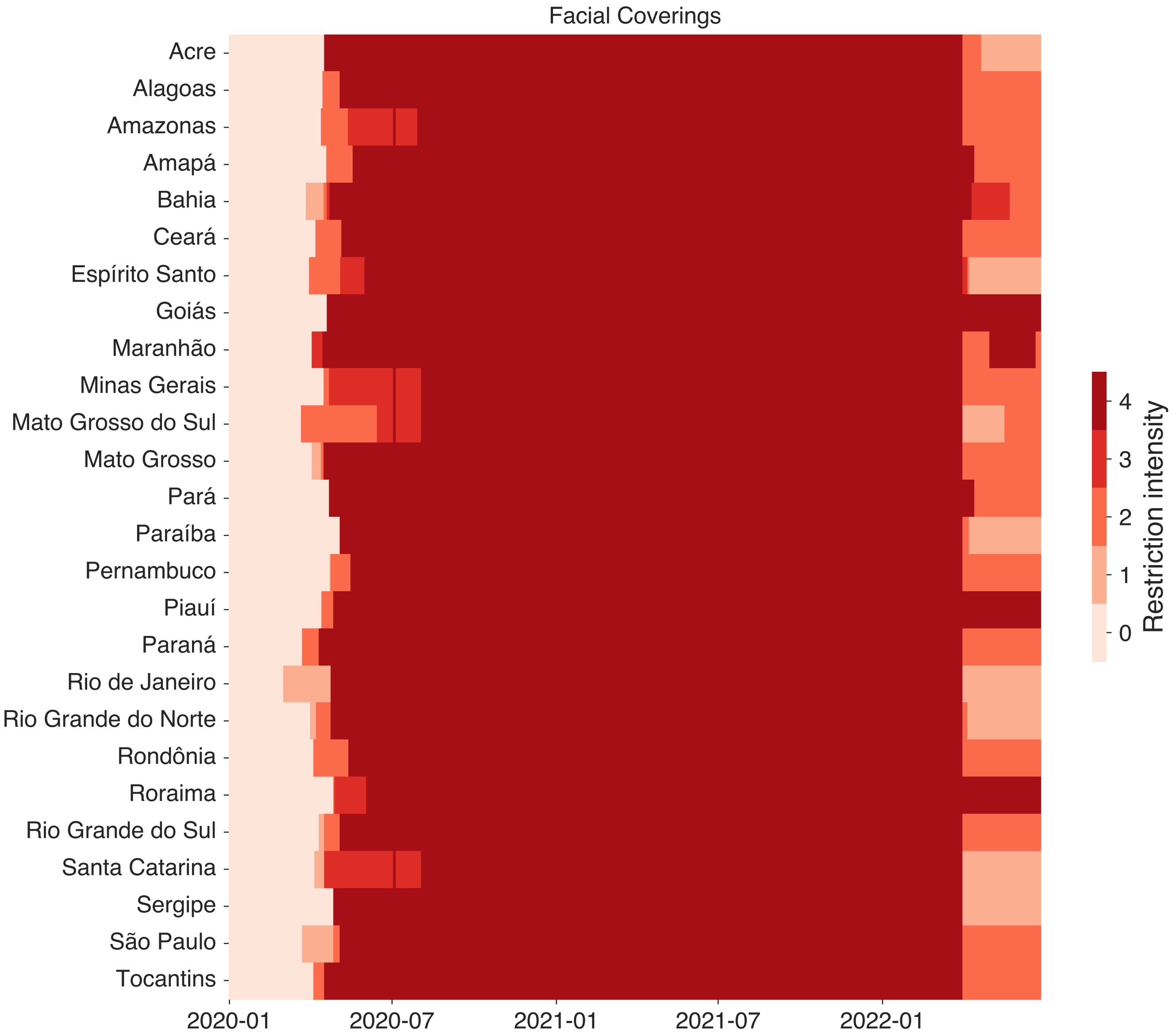

Figure S8. Facial covering non-pharmaceutical interventions among the Brazilian states. The heatmap displays the temporal evolution of the implementation of facial coverings interventions for all Brazilian states from January 2020 to June 2022. The color code stands for the degree of restriction: "no policy" (0), "recommended" (1), "required in some specified shared/public spaces outside the home with other people present, or some situations when social distancing not possible" (2), "required in all shared/public spaces outside the home with other people present or all situations when social distancing not possible" (3), and "required outside the home at all times regardless of location or presence of other people" (4).

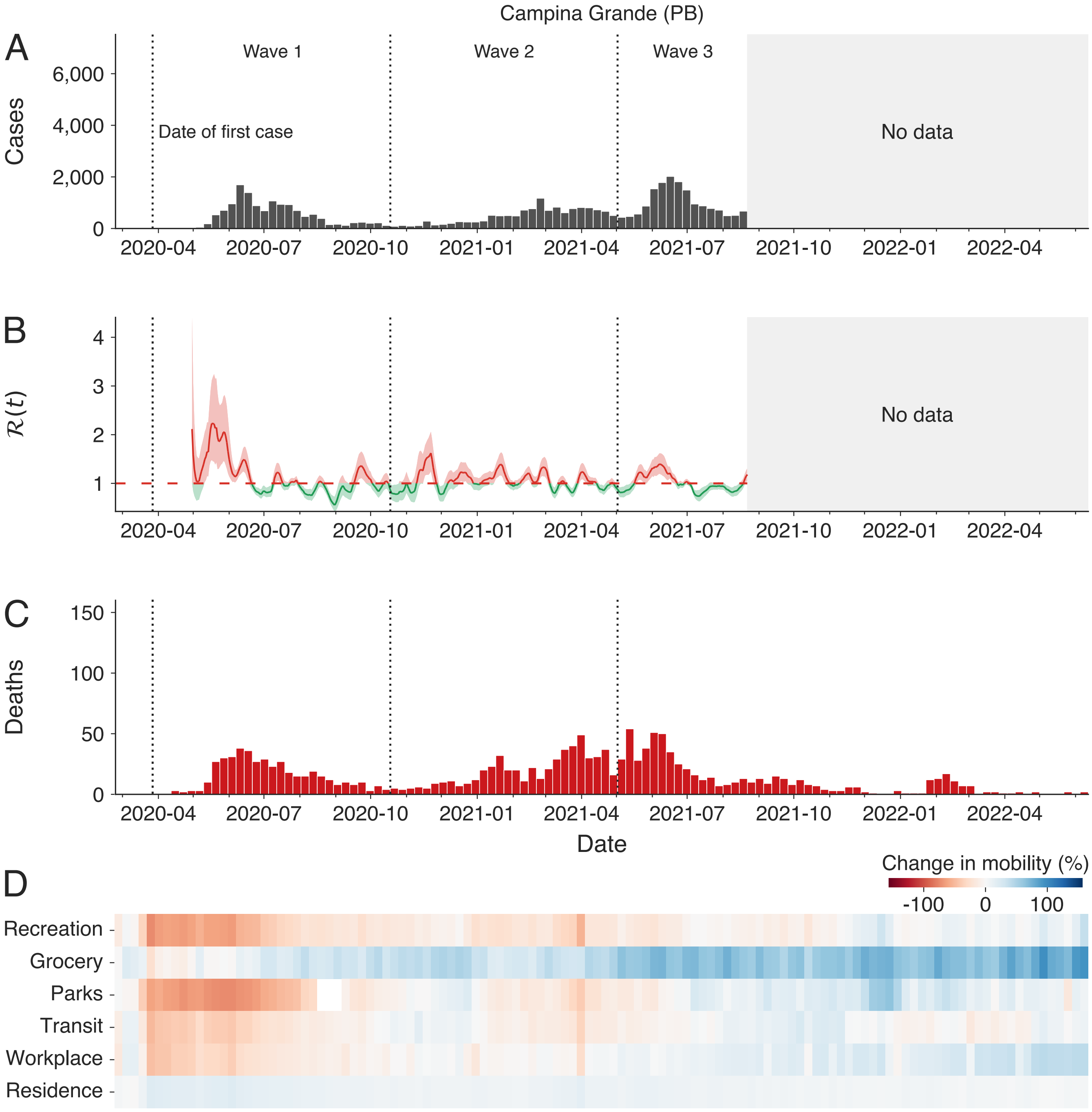

Figure S9. Indicators of the COVID-19 pandemic in Campina Grande (PB). (A) Weekly number of confirmed cases of COVID-19 between 22 March 2020 and 22 August 2021. (B) Instant reproduction number $\mathscr{R}(t)$ from 30 April 2020 to 22 August 2021. Shaded regions represent the 95% confidence intervals, and the dashed horizontal line indicates the epidemic threshold $\mathscr{R}(t) = 1$. (C) Weekly COVID-19 death toll between 22 March 2020 and 12 June 2022. In the previous panels, vertical dashed lines delineate the identified waves of COVID-19 cases. (D) Temporal heatmap illustrating the changes in mobility related to Google users' visiting patterns to places categorized into six groups (recreation, grocery, parks, transit, workplace, and residence) compared to baselines estimated using pre-pandemic levels. Blue shades indicate an increase in the visitation to a place category, while red shades indicate a reduction.

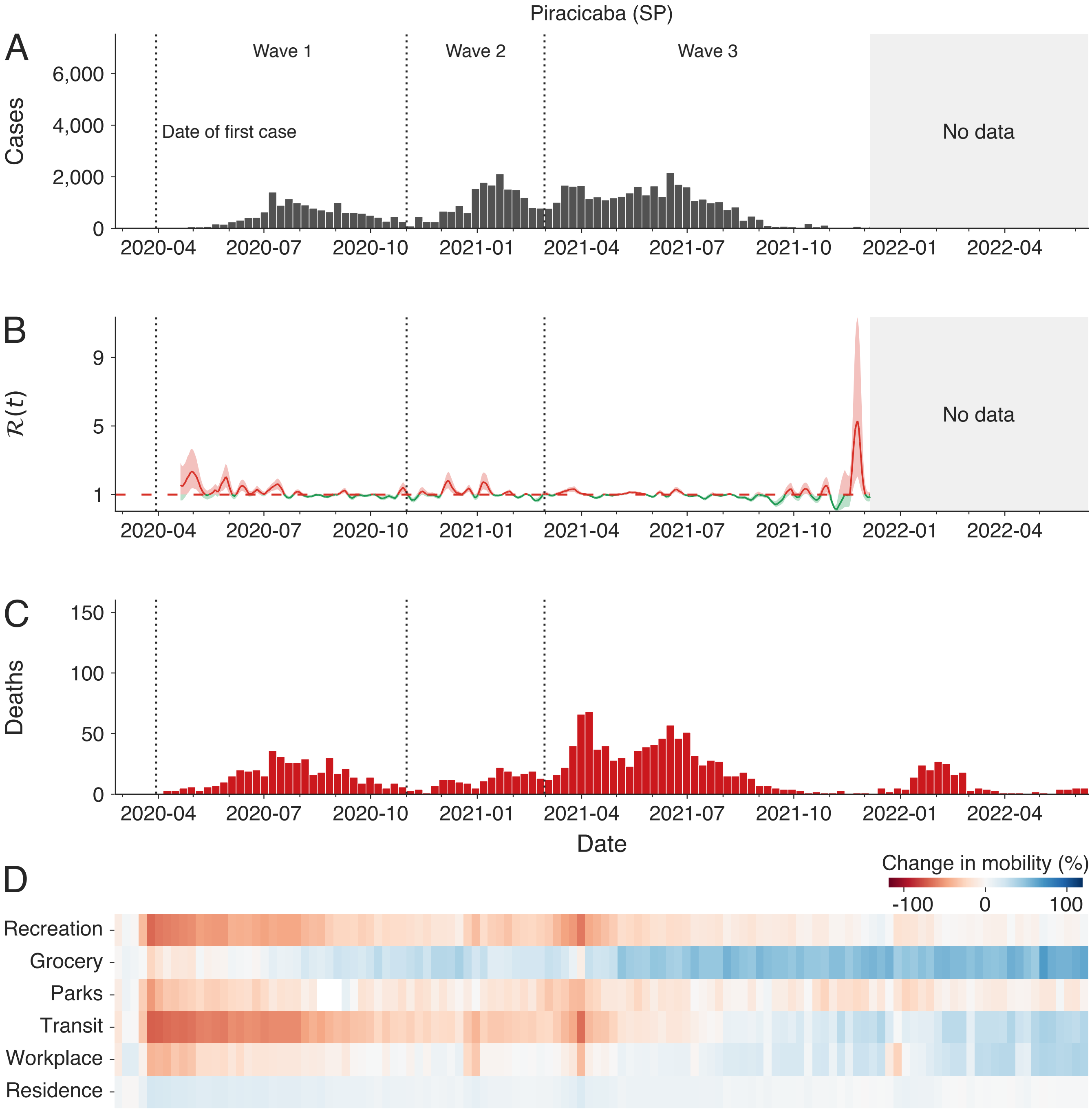

Figure S10. Indicators of the COVID-19 pandemic in Piracicaba (SP). (A) Weekly number of confirmed cases of COVID-19 between 29 March 2020 and 12 December 2021. (B) Instant reproduction number $\mathscr{R}(t)$ from 20 April 2020 to 6 December 2021. Shaded regions represent the 95% confidence intervals, and the dashed horizontal line indicates the epidemic threshold $\mathscr{R}(t) = 1$. (C) Weekly COVID-19 death toll between 29 March 2020 and 12 June 2022. In the previous panels, vertical dashed lines delineate the identified waves of COVID-19 cases. (D) Temporal heatmap illustrating the changes in mobility related to Google users' visiting patterns to places categorized into six groups (recreation, grocery, parks, transit, workplace, and residence) compared to baselines estimated using pre-pandemic levels. Blue shades indicate an increase in the visitation to a place category, while red shades indicate a reduction.

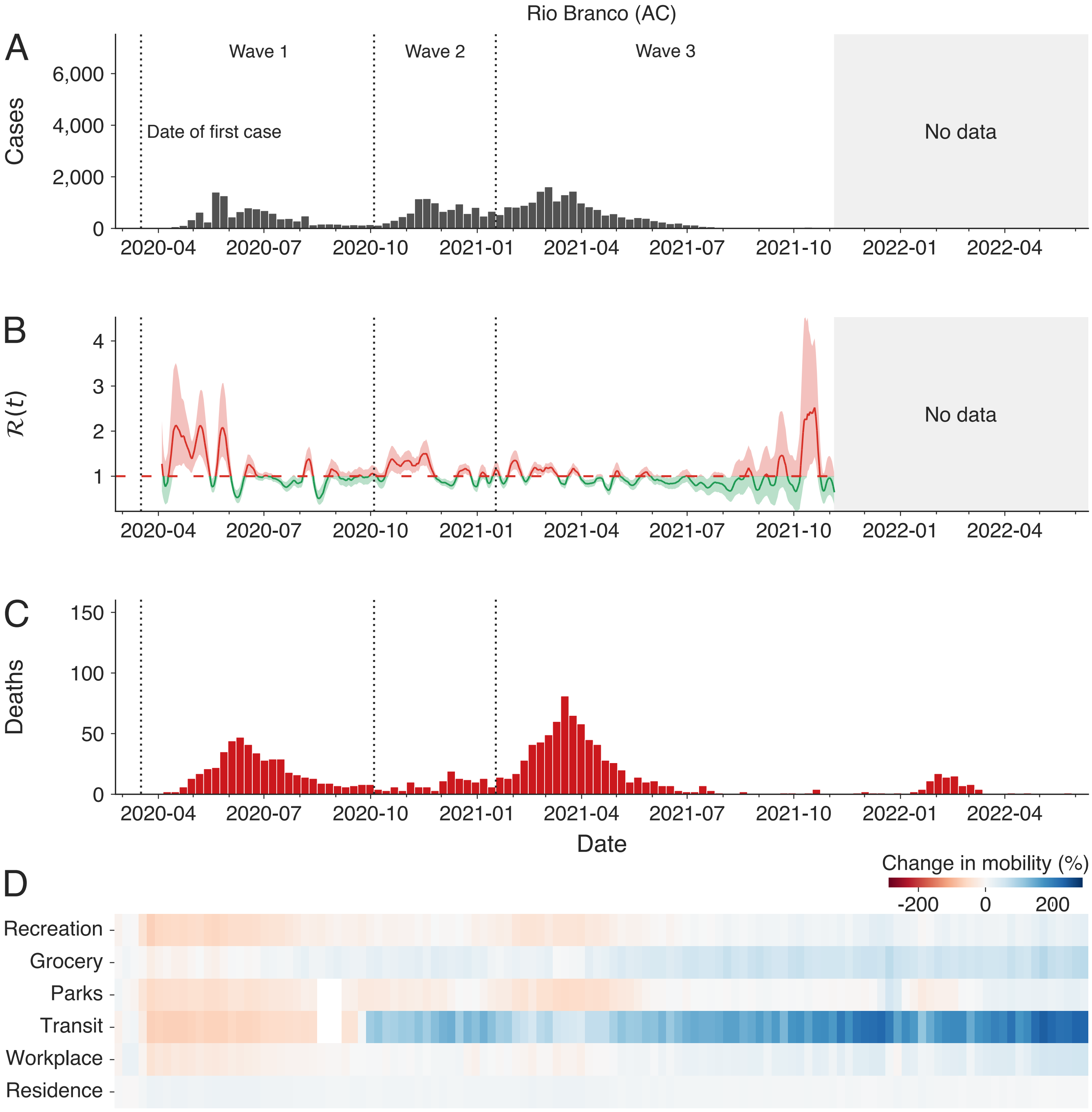

Figure S11. Indicators of the COVID-19 pandemic in Rio Branco (AC). (A) Weekly number of confirmed cases of COVID-19 between 15 March 2020 and 7 November 2021. (B) Instant reproduction number $\mathscr{R}(t)$ from 4 April 2020 to 5 November 2021. Shaded regions represent the 95% confidence intervals, and the dashed horizontal line indicates the epidemic threshold $\mathscr{R}(t) = 1$. (C) Weekly COVID-19 death toll between 15 March 2020 and 12 June 2022. In the previous panels, vertical dashed lines delineate the identified waves of COVID-19 cases. (D) Temporal heatmap illustrating the changes in mobility related to Google users' visiting patterns to places categorized into six groups (recreation, grocery, parks, transit, workplace, and residence) compared to baselines estimated using pre-pandemic levels. Blue shades indicate an increase in the visitation to a place category, while red shades indicate a reduction.

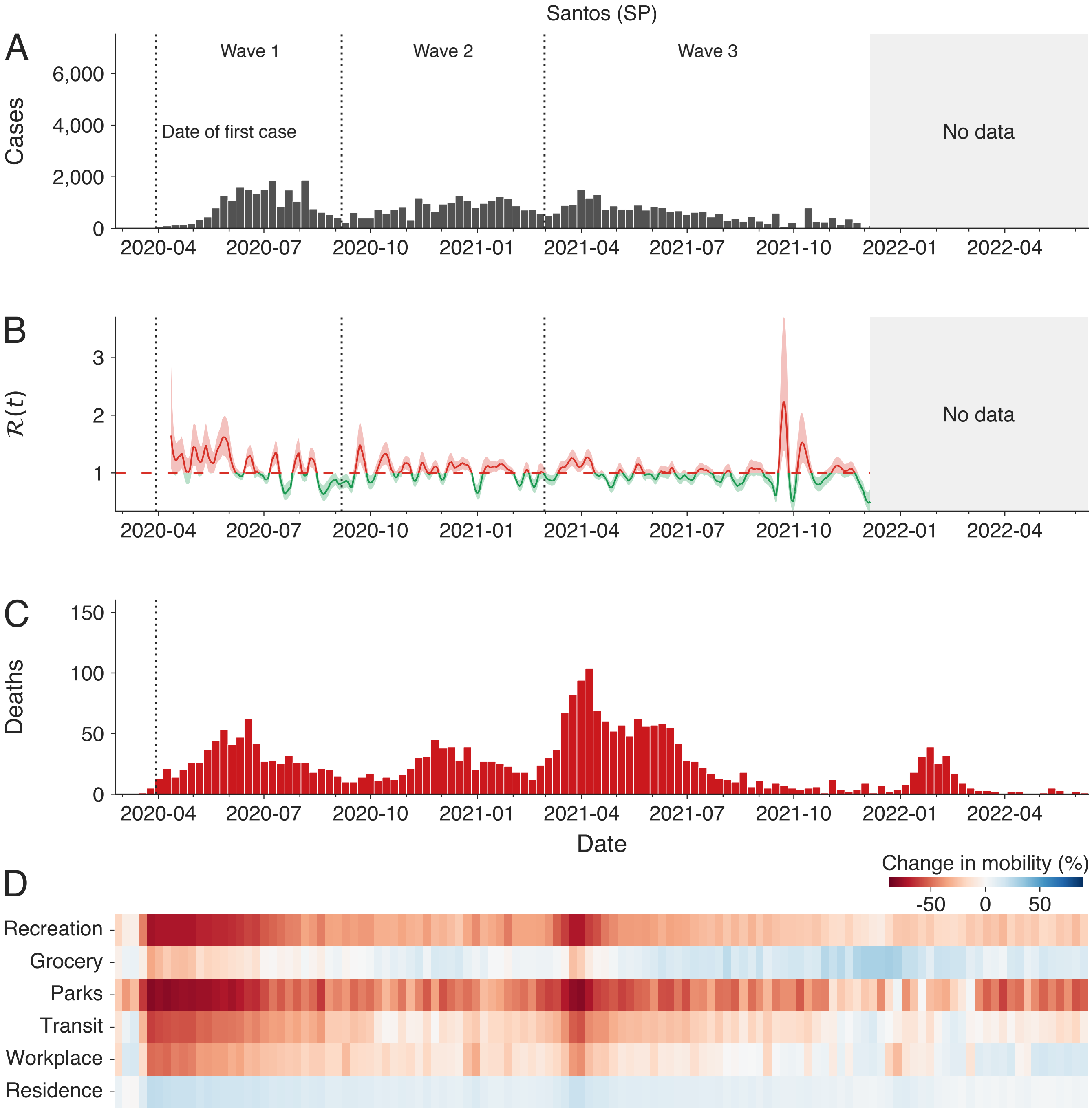

Figure S12. Indicators of the COVID-19 pandemic in Santos (SP). (A) Weekly number of confirmed cases of COVID-19 between 29 March 2020 and 12 December 2021. (B) Instant reproduction number $\mathscr{R}(t)$ from 12 April 2020 to 6 December 2021. Shaded regions represent the 95% confidence intervals, and the dashed horizontal line indicates the epidemic threshold $\mathscr{R}(t) = 1$. (C) Weekly COVID-19 death toll between 29 March 2020 and 12 June 2022. In the previous panels, vertical dashed lines delineate the identified waves of COVID-19 cases. (D) Temporal heatmap illustrating the changes in mobility related to Google users' visiting patterns to places categorized into six groups (recreation, grocery, parks, transit, workplace, and residence) compared to baselines estimated using pre-pandemic levels. Blue shades indicate an increase in the visitation to a place category, while red shades indicate a reduction.

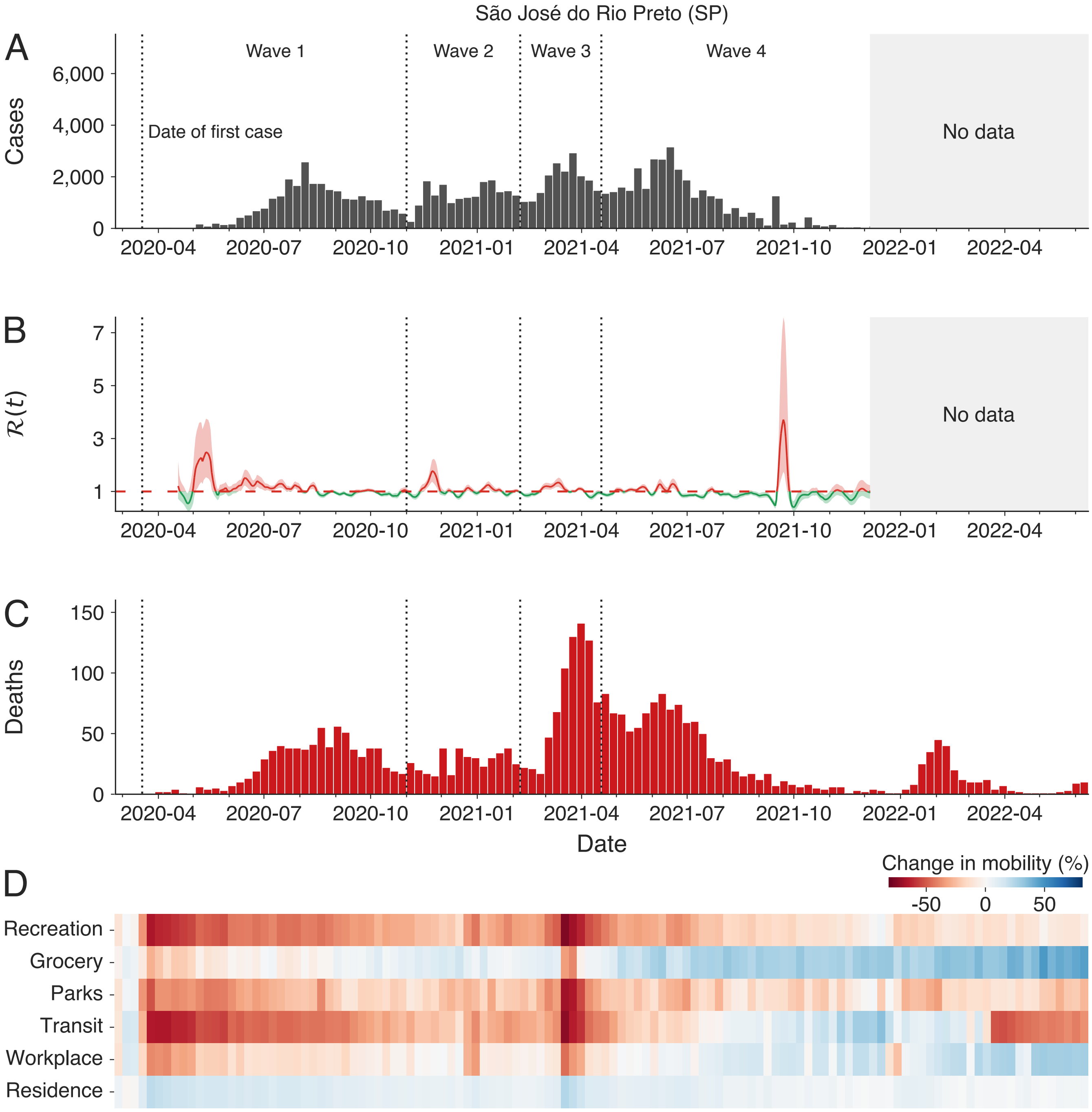

Figure S13. Indicators of the COVID-19 pandemic in São José do Rio Preto (SP). (A) Weekly number of confirmed cases of COVID-19 between 5 March 2020 and 12 December 2021. (B) Instant reproduction number $\mathscr{R}(t)$ from 18 April 2020 to 6 December 2021. Shaded regions represent the 95% confidence intervals, and the dashed horizontal line indicates the epidemic threshold $\mathscr{R}(t) = 1$. (C) Weekly COVID-19 death toll between 15 March 2020 and 12 June 2022. In the previous panels, vertical dashed lines delineate the identified waves of COVID-19 cases. (D) Temporal heatmap illustrating the changes in mobility related to Google users' visiting patterns to places categorized into six groups (recreation, grocery, parks, transit, workplace, and residence) compared to baselines estimated using pre-pandemic levels. Blue shades indicate an increase in the visitation to a place category, while red shades indicate a reduction.

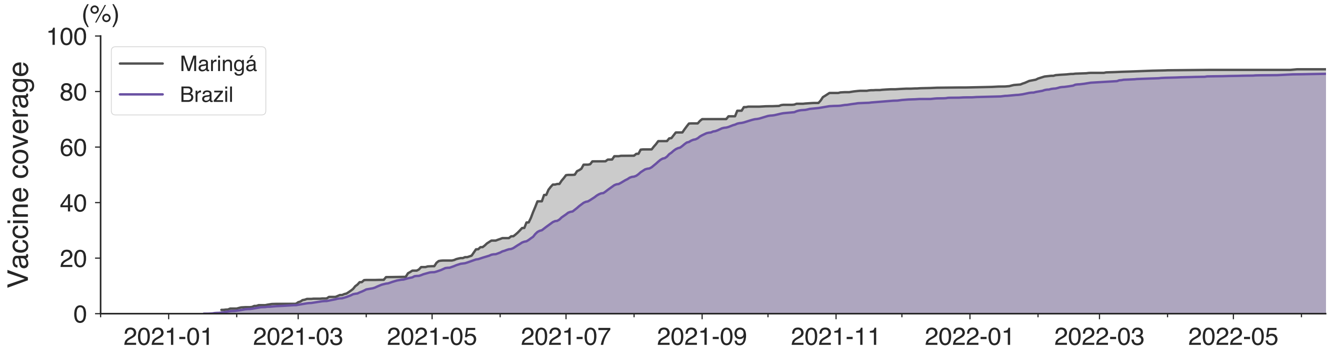

Figure S14. Comparison of the evolution of the vaccine coverage between Maringá and Brazil. The two curves represent the percentage of population immunized with the first dose of a COVID-19 vaccine in Maringá (gray curve) and in Brazil (purple curve) from the beginning of 2021 to 12 June 2022.

Table S1. Deaths and percentage of deaths caused by COVID-19 in each epidemic wave in Maringá. The table shows information about the deaths and percentage of deaths grouping the population by age and gender in each epidemic wave that took place in the city. The percentage of deaths in each wave may not sum up to a hundred per cent because the total number of casualties can include deaths classified as undefined gender.

Gender	Age group	Wave 1	Wave 2	Wave 3	Wave 4	Wave 5	Wave 6	All waves
All	All	184 deaths	250 deaths	572 deaths	686 deaths	122 deaths	33 deaths	1,847 deaths
	0 - 19	1.09%	0.00%	0.52%	0.15%	1.64%	0.00%	0.43%
	20 - 29	1.09%	0.00%	0.52%	1.17%	0.00%	3.03%	0.76%
	30 - 39	4.89%	2.00%	4.37%	4.96%	0.82%	0.00%	4.01%
	40 - 49	2.17%	3.20%	9.62%	13.56%	1.64%	3.03%	8.83%
	50 - 59	10.87%	8.40%	14.86%	21.87%	4.10%	0.00%	15.21%
	$\geq$ 60	79.89%	86.40%	70.10%	58.31%	91.80%	93.94%	70.76%
Female	All	81 deaths	101 deaths	242 deaths	262 deaths	45 deaths	17 deaths	748 deaths
	All	44.02%	40.40%	42.31%	38.19%	36.89%	51.52%	40.50%
	0 - 19	0.54%	0.00%	0.35%	0.00%	1.64%	0.00%	0.27%
	20 - 29	0.54%	0.00%	0.17%	0.58%	0.00%	3.03%	0.38%
	30 - 39	1.09%	0.40%	1.75%	1.75%	0.00%	0.00%	1.35%
	40 - 49	0.54%	0.40%	1.75%	3.50%	0.82%	0.00%	2.00%
	50 - 59	4.35%	3.20%	6.64%	7.29%	0.82%	0.00%	5.68%
	$\geq$ 60	36.96%	36.40%	31.64%	25.07%	33.61%	48.48%	30.81%
Male	All	103 deaths	149 deaths	330 deaths	422 deaths	77 deaths	16 deaths	1,097 deaths
	All	55.98%	59.60%	57.69%	61.52%	63.11%	48.48%	59.39%
	0 - 19	0.54%	0.00%	0.17%	0.15%	0.00%	0.00%	0.16%
	20 - 29	0.54%	0.00%	0.35%	0.58%	0.00%	0.00%	0.38%
	30 - 39	3.80%	1.60%	2.62%	3.21%	0.82%	0.00%	2.65%
	40 - 49	1.63%	2.80%	7.87%	10.06%	0.82%	3.03%	6.82%
	50 - 59	6.52%	5.20%	8.22%	14.29%	3.28%	0.00%	9.42%
	$\geq$ 60	42.93%	50.00%	38.46%	33.24%	58.20%	45.45%	39.96%

Table S2. Metrics associated with the COVID-19 confirmed cases and the instantaneous reproduction number in Maringá, five cities of similar size, and Brazil. The table shows information about the estimated 2021 population, number of total cases, number of weekly cases per 100,000 inhabitants, peak number of weekly cases per 100,000 inhabitants, maximum value of the instantaneous reproduction number $\mathscr{R}_{\text{max}}$, proportion of days with $\mathscr{R}(t) > 1$, and average number of consecutive days with $\mathscr{R}(t) > 1$ ($\pm$ one standard deviation). The time window used to calculate these metrics spanned from the date of first case to 22 August 2021, when we have available data for all cities and Brazil.

Place	Population	Total cases	Weekly cases per 100k	Peak weekly cases per 100k	$\mathscr{R}_{\text{max}}(t)$	Proportion of days with $\mathscr{R}(t) > 1$	Average number of consecutive days with $\mathscr{R}(t) > 1$
Maringá	436,472	62,327	192	500	2.47	0.59	16.56 $\pm$9.64
Campina Grande	413,830	42,778	141	488	2.23	0.52	14.59$\pm$13.70
Piracicaba	410,275	64,520	179	527	2.35	0.52	12.14$\pm$10.78
Rio Branco	419,452	38,206	107	384	2.13	0.44	11.25$\pm$10.70
Santos	433,991	62,487	164	431	1.65	0.55	13.14$\pm$12.05
São José do Rio Preto	469,173	96,187	229	672	2.49	0.53	13.84$\pm$13.55
Brazil	213,317,639	20,595,014	125	253	4.27	0.59	19.38$\pm$22.46

Table S3. Metrics associated with the deaths caused by COVID-19 in Maringá, five cities of similar size, and Brazil. The table shows information about the estimated 2021 population, number of total deaths, number of weekly deaths per 100,000 inhabitants, and peak number of weekly deaths per 100,000 inhabitants for Maringá, five cities of similar size, and Brazil. The time window used to calculate these metrics spanned from the date of the first death to 12 June 2022 (when information was available for all cities and Brazil).

Place	Population	Total deaths	Weekly deaths per 100k	Peak weekly deaths per 100k
Maringá	436,472	1,847	3.73	23.37
Campina Grande	413,830	1,630	1.98	13.05
Piracicaba	410,275	1,837	2.25	16.57
Rio Branco	419,452	1,443	1.73	19.31
Santos	433,991	2,837	3.28	23.96
São José do Rio Preto	469,173	3,363	3.60	30.05
Brazil	213,317,639	681,934	1.61	11.20

Table S4. Metrics associated with deaths caused by COVID-19 for each age group in Maringá and Brazil. The table shows information, for each age group, about the total deaths, percentage of deaths, percentage of female deaths, percentage of male deaths, deaths per 100,000 inhabitants, female deaths per 100,000 inhabitants, and male deaths per 100,000 inhabitants in Maringá and Brazil. The 2021's demographic structure of each region was estimated by using the 2010's age and gender structures further rescaled by the ratio between the population estimation of 2021 and the population in 2010. The percentage of deaths for female and male population does not sum up to a hundred per cent because the total number of casualities include deaths classified as undefined gender.

Age group		0-19	20-29	30-39	40-49	50-59	≥ 60
Total deaths	Maringá	8	14	74	163	281	1,307
	Brazil	4,650	6,422	22,976	53,044	93,487	478,600
Percentage of deaths	Maringá	0.43%	0.76%	4.01%	8.83%	15.21%	70.76%
	Brazil	0.71%	0.97%	3.49%	8.05%	14.18%	72.61%
Percentage of deaths (female)	Maringá	0.27%	0.38%	1.36%	2.01%	5.69%	30.84%
	Brazil	0.31%	0.47%	1.42%	3.15%	5.69%	33.01%
Percentage of deaths (male)	Maringá	0.16%	0.38%	2.66%	6.83%	9.43%	40.00%
	Brazil	0.36%	0.50%	2.07%	4.89%	8.49%	39.59%
Deaths per 100k people	Maringá	5.58	13.83	88.94	203.73	462.14	2,016.82
	Brazil	5.91	14.95	62.00	170.73	405.90	1,858.61
Deaths per 100k people (female)	Maringá	8.65	16.77	70.77	105.68	387.15	1,923.23
	Brazil	5.91	16.18	55.18	144.86	346.22	1,701.58
Deaths per 100k people (male)	Maringá	5.04	17.03	149.64	413.89	769.16	3,149.65
	Brazil	6.63	17.26	84.12	240.05	573.06	2,548.46
